# Elevated Levels of Plasma Collagen Triple Helix Repeat Containing 1 (CTHRC1) Are Strongly Associated with eGFR and Albuminuria in Chronic Kidney Disease

**DOI:** 10.3390/medicina59040651

**Published:** 2023-03-24

**Authors:** Yeldar Baiken, Zhannur Markhametova, Assem Ashimova, Ainur Zhulamanova, Assem Nogaibayeva, Larissa Kozina, Bakhyt Matkarimov, Bauyrzhan Aituov, Abduzhappar Gaipov, Askhat Myngbay

**Affiliations:** 1 School of Engineering and Digital Sciences, Nazarbayev University, Astana 010000, Kazakhstan; 2 PI National Laboratory Astana, Nazarbayev University, Astana 010000, Kazakhstan; 3 School of Sciences and Humanities, Nazarbayev University, Astana 010000, Kazakhstan; 4 Department of Medicine, Nazarbayev University School of Medicine, Astana 020000, Kazakhstan; 5 Department of Education, LLP BBNura, Astana 010000, Kazakhstan; 6 Department of Laboratory Diagnostics, National Scientific Medical Center, Astana 010000, Kazakhstan; 7 Center for BioEnergy Research LLP, Astana 010000, Kazakhstan; 8 Clinical Academic Department of Internal Medicine, CF “University Medical Center”, Astana 010000, Kazakhstan

**Keywords:** chronic kidney disease, CKD, kidney disease, glomerulonephritis, CTHRC1

## Abstract

*Background*: Chronic kidney disease (CKD) has various etiologies, making it impossible to fully understand its complex pathophysiology. Elevated levels of plasma creatinine, proteinuria, and albuminuria and declined eGFR are traits observed in CKD patients. The current study attempts to highlight the collagen triple helix repeat containing 1 (CTHRC1) protein as a putative blood biomarker for CKD in addition to existing recognized indicators of CKD progression. *Methods*: A total of 26 CKD patients and 18 healthy controls were enrolled in this study. Clinical characteristics and complete blood and biochemical analyses were collected, and human ELISA kits were used to detect possible CKD biomarkers. *Results*: The study’s findings showed that CTHRC1 correlates with key clinical markers of kidney function such as 24 h urine total protein, creatinine, urea, and uric acid. In addition, CTHRC1 demonstrated a strong significant difference (*p* ≤ 0.0001) between the CKD and control group. *Conclusions*: Our research demonstrates that the plasma level of CTHRC1 can distinguish between those with CKD and healthy patients. Plasma CTHRC1 levels may aid in the diagnosis of CKD given the current state of knowledge, and these results call for further investigation in a wider, more diverse patient group.

## 1. Introduction

Chronic kidney disease (CKD) has a varied etiology, and comprehending its complicated pathophysiology is inherently difficult [1]. Endothelial and tissue injuries are linked to the release of specific mediators that may initiate the inflammatory cascade in the pathogenesis of acute kidney injury (AKI) and CKD. Chronic low-grade inflammation is a hallmark of CKD. Numerous cytokines and chemokines have been linked to CKD complications and have been found in the plasma and urine of patients who are in the early stages of the disease. Renal fibrosis is caused by several pathological events, including an influx of inflammatory cells, the activation and proliferation of fibroblasts, the deposition of the extracellular matrix (ECM) (i.e., collagen, fibronectin, proteoglycan), and the replacement of host tissue by fibrotic tissue. These events lead to the malfunction or loss of tubule functions, loss of peritubular capillaries, and remodeling of tissue architecture, eventually resulting in renal failure [2,3].

The main causes of renal fibrosis are considered to be prolonged inflammation, excessive accumulation of ECM components, and initial renal injury, overall leading to renal scar formation and end-stage renal disease (ESRD) [4,5]. It is well known that the TGFβ signaling pathway is one of the key mediators of renal fibrosis. Active TGFβ1 binds to the TGFβ receptor (TβRII), consequently activating the type I TGFβ receptor and Smads (Smad2 and −3). Smad2, Smad3, and Smad4 form a complex that translocates into the nucleus. Furthermore, this Smad complex induces α-smooth muscle actin (αSMA), collagens, and inhibitory Smad7 by acting as a transcriptional regulator of target genes [6]. 

The involvement of the both canonical and non-canonical TGFβ/Smad signaling pathways in renal fibrosis has been thoroughly investigated. Edeling et al. showed that TGFβ/Smad interacts with other signaling pathways, such as Wnt/β-catenin, Jagged1/Notch, and Hedgehog, during epithelial differentiation, myofibroblast transformation, and proliferation [7]. 

Collagen triple helix repeat containing 1 (CTHRC1) is a secreted, glycosylated protein that is normally expressed in mesenchymal connective tissue during embryonic development. The overexpression of CTHRC1 in adult mesenchyme-derived cells is associated with enhanced cell motility and the invasiveness of cancer metastases [8]. It has been demonstrated that CTHRC1 interacts with the TGFβ signaling pathway during vascular remodeling after injury. Furthermore, Pyagay et al. demonstrated that bone morphogenetic protein (BMP) and TGFβ can regulate the transcription of CTHRC1 through Smad proteins [9]. It was also shown that CTHRC1 inhibits the activation of Smad2/3, consequently leading to the production of fibrous protein [10].

Numerous studies demonstrate that the Wnt/β-catenin signaling pathway is crucial for the progression of renal fibrosis, podocyte injury, and proteinuria [11,12,13,14]. It is known that the Wnt signaling pathway consists of the canonical (Wnt/β-catenin pathway), non-canonical (Wnt/planar cell polarity pathway (Wnt/PCP pathway)), and Wnt/calcium (Wnt/Ca2+ pathway) pathways [12,15].

According to a report, CTHRC1 stabilizes the formation of the WNT5A-ROR2 complex, allowing it to specifically activate the planar cell polarity pathway of Wnt signaling [16]. CTHRC1 interacts with multiple FZD receptors and modulates cascades of the Wnt/PCP pathway [16]. Wnt5a is one of the Wnt ligands that has certain functions in regulating kidney morphogenesis [17]. A study of 41 patients with ESRD showed that the upregulation of Wnt5a and β-catenin can lead to vascular calcification [18], and another study showed that a loss of function of WNT5a leads to kidney malformation [19].

Studies have shown that CTHRC1 alters cell adhesion via modulating the production of molecules, including integrin and MMPs, allowing cells to move more freely and increasing tumor spread and invasion. Moreover, it was shown that CTHRC1 has a huge impact on the formation of fibrosis in the liver [20] via the modulation of the TGF-β signaling pathway. CTHRC1 suppresses TGF signaling by speeding up the degradation of phospho-Smad3 via a proteosomal mechanism [21]. CTHRC1 has varied cellular localization and methods of action in different cells and microenvironments, which is worth highlighting [22]. A recent study [23] demonstrated that the patients’ overall survival time was considerably shortened (*p* > 0.05 in Kaplan–Meier plotter) when their CTHRC1 transcriptional level was greater in various cancers.

Taking into account the crucial roles of TGFβ signaling, as well as canonical and non-canonical pathways in CKD, we hypothesized that CTHRC1 can have a certain role in renal dysfunction. To address this question, a randomized cross-sectional pilot study was performed. In this study, we aimed to analyze the levels of CTHRC1 in the peripheral blood of CKD patients and to test CTHRC1′s association with other markers of CKD. 

## 2. Materials and Methods

### 2.1. Study Population and Design

This project is a prospective observational validation study. The 26 patients with established diagnoses of CKD were recruited from the “National Scientific Medical Center” hospital while they were admitted to the internal medicine department. Eighteen volunteers were enrolled from the outpatient clinics. All of the included participants had stable clinical circumstances and no active malignancy, autoimmune, cardiovascular, neuropsychiatric, or inflammatory illnesses. Samples were collected to the hospital in a time period of 14 months. The approximate time interval averaged 2–3 patients per month. Up until the conclusion of the study, the laboratory researchers were blinded to data revealing patients’ clinical outcomes. Independent blood and urine samples were taken without taking the patients’ prognosis into account. For each participant, blood metabolic profiles were acquired, including those for serum creatinine, serum urea, glucose, uric acid, total protein, AST, ALT, lipid profiles, and protein fractions. A 24 h urinalysis with values for the urine protein, sodium, potassium, uric acid, and creatinine was also defined at the time of registration.

The following inclusion criteria for CKD patient group were used: according to KDIGO classification, CKD stages varied between 1 and 3; enrolled patients’ age was between 18 and 65 years old; and proteinuria levels were greater than 300 mg/24 h. 

CKD patients with the following characteristics were excluded: glomerular filtration rate (GFR) < 15 mL/min; females at any stage of pregnancy; patients with accompanying diseases such as diabetes mellitus (Type I and II), any type of cancer, infectious diseases, and any other inflammatory and autoimmune diseases specifically liver fibrosis, rheumatoid arthritis, and other life-treating comorbidities/conditions. The diagnosis of CKD was based on the levels of proteinuria and was not confirmed by kidney biopsy. CKD-EPI Creatinine Equation (2021) was used to estimate GFR and CKD stages, as recommended by the National Kidney Foundation [24]. CKD patients (38.8 ± 14.5 (mean ± SD) years of age) were classified into 3 groups according to eGFR: CKD stage 1 (>90 mL/min/173 m^2^, *n* = 13), CKD stage 2 (60–89 mL/min/173 m^2^, *n*= 7), and CKD stage 3 (30–59 mL/min/173 m^2^, *n*= 6). The control group consisted of 18 subjects; 34.7 ± 8.05 years of age; without a medical history of renal disease, and with eGFR more than 60 mL/min/1.73 m^2^.

### 2.2. Ethics Statement

All work within the project was conducted in accordance with international and national legal and ethical principles. All study procedures were approved by the Nazarbayev University Institutional Review Ethics Committee on 25 February 2020 (NU-IREC 208/06122019). Written informed consent was obtained from all participants. 

### 2.3. Blood Sampling

Peripheral venous blood samples were collected from each participant in the morning on an empty stomach. Each participant was identified before to sampling and given all the information required to be entered into the laboratory information system. The puncture was performed while the participants were seated; to prevent venous stasis, the puncture site was cleansed with 70% alcohol, and the tourniquet was applied for less than three minutes. To ensure the traceability between the patient and the samples, each tube was assigned a distinct bar code. Blood samples were collected on etheylenediaminetetraacetic acid (EDTA) tubes for complete blood count (CBC), and lithium heparin tubes for biochemical analysis. EDTA plasma was separated within 30 to 90 min of sample collection; samples were centrifuged at RT for 15 min. at 2200 g. After the CBC analysis, plasma were stored at −40 °C until the CTHRC1 ELISA test was performed.

### 2.4. Laboratory Measurements

All laboratory measurements were performed in the laboratory at the “National Scientific Medical Center” hospital. Plasma creatinine, urea, and CRP concentrations were measured using an automated biochemical analyzer (Cobas 6000) with the commercially available kits. CBC analysis was completed using a Sysmex-XN 3000 analyzer. CTHRC1 levels were measured in the Biology department at the PI “National Laboratory Astana”, Nazarbayev University using Human CTHRC1 SimpleStep ELISA kit (cat number: ab274399, Abcam, Cambridge, UK), according to the manufacturers’ protocol. 

### 2.5. Enzyme Linked Immunoassay (ELISA)

Human CTHRC1 SimpleStep ELISA kit (cat number: ab274399, Abcam), TNF alpha ELISA Kit (cat number: ab46087, Abcam), Human MMP9 Elisa kit (cat number: ab100610, Abcam), and Human MMP2 Elisa kit (cat number: ab100606, Abcam) were used to detect proteins in plasma samples. First, stock standards were prepared according to the Human CTHRC1 SimpleStep ELISA kit protocol. The 96-well plate strips included with the kit were supplied and ready to use. All materials and reagents were equilibrated and prepared at RT prior to use. Sample and standards were diluted to ½ for CTHRC1, ½ for TNF alpha, 1/10 for MMP2, and 1/50 for MMP9 with a Sample Diluent buffer. Fifty microliters of each standard and sample was added into appropriate wells. Then, 50 µL of the Antibody Cocktail was added to each well. The plate was sealed and incubated for 1 h at RT on a plate shaker set to 400 rpm. Each well was washed with 3 × 350 µL 1× Wash Buffer PT. The wells were washed by aspirating and then dispensing 350 µL 1× Wash Buffer PT into each well. Next, 100 µL of TMB Development Solution (provided with the kit) was added to each well and incubated for 10 min in the dark on a plate shaker set to 400 rpm. The reaction was stopped by adding 100 µL of Stop Solution to each well. The absorbance of each plate was measured at 450 nm with the reference wavelength of 620 nm using the VarioScan Flash microplate reader (Thermo Fisher Scientific, Waltham, MA, USA) [25]. 

### 2.6. Statistical Analysis

The median and interquartile ranges (IQR) or the mean with standard deviation (SD) were used to summarize patient data. Student’s *t*-test was used to compare normally distributed data; otherwise, the Mann–Whitney U-test was used. For qualitative variables, the Pearson’s correlation test was used. The link between two continuous variables was described by non-parametric Spearman correlation coefficients. When employed as continuous variables, CTHRC1 levels had a skewed distribution and underwent log transformation. All reported *p*-values were two-tailed, and significance was set at *p* value ≤ 0.05. For statistical analysis and visual representations, GraphPad Prism version 8.0.1 for Windows was used (GraphPad Software, La Jolla, CA, USA).

## 3. Results

### 3.1. Subject Characteristics

The analysis of a 24 h urine collection is the gold standard for quantifying proteinuria. Expectedly, the median protein levels in 24 h urine collection for the CKD and control groups were 1.66 g/24 h and 0.09 g/24 h, respectively (Table 1). The mean eGFR rates were significantly reduced (91.12 ± 34.2 in CKD and 109.76 ± 12.4 in control) in the CKD group compared with the control (*p* value = 0.0279) (Table 1). The mean creatinine concentration was 90.55 ± 38.1 umol/L in the CKD group. Complete blood count analysis revealed significance only in WBCs (*p* = 0.0059), among which the difference in neutrophils (*p* = 0.0089) and monocytes (*p* = 0.0068) between the CKD and control groups was significant (Table 1). Lipid levels in the blood (mainly total cholesterol, triglycerides, and LDLs) had significant variance between the CKD and control group (Table 1). ELISA tests on TNF alpha, MMP2, and MMP9 did not show any significant differences between the control and CKD groups (data not shown), whereas CTHRC1 concentrations were significantly higher in the CKD group (*p* < 0.0001).

### 3.2. The Relationship between CTHRC1 and CKD Indicators

**Figure 1:**
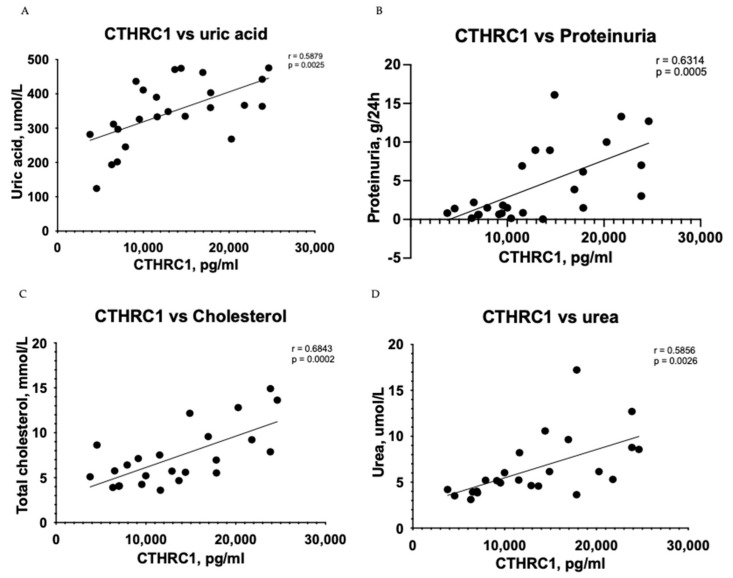

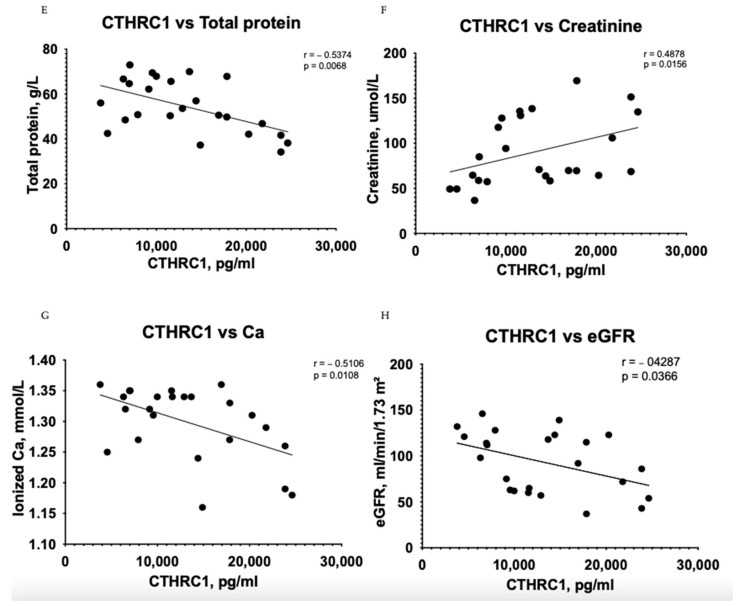
Correlation between plasma levels of CTHRC1 and notable clinical measures in CKD patients. The relationship between variables was evaluated using Pearson’s correlation test. Trend lines indicate linear correlation. Pearson’s (r) coefficient and the corresponding *p*-values are shown on each plot. Scatter plot graphs illustrating correlation between (**A**) CTHRC1 and uric acid, (**B**) CTHRC1 and total protein collected in 24 h in urine, (**C**) CTHRC1 and cholesterol, (**D**) CTHRC1 and urea, (**E**) CTHRC1 and plasma total protein levels, (**F**) CTHRC1 and creatinine (CTHRC1 and creatinine parameters were analyzed via Spearman’s rank correlation test), (**G**) CTHRC1 and ionized calcium levels, (**H**) CTHRC1 and eGFR.

The Pearson correlation test demonstrated that CTHRC1 correlates with many essential clinical markers of kidney function, including 24 h collected total protein levels in urine (r = 0.6314, *p* = 0.0005) (Figure 1A), uric acid (r = 0.5879, *p* = 0.0025) (Figure 1B), urea (r = 0.5856, *p* = 0.0026) (Figure 1D), total protein levels (r = 0.5374, *p* = 0.0068) (Figure 1E) and eGFR (r = −0.4287, *p* = 0.0366) (Figure 1H). CTHRC1 and creatinine (r = 0.4878, *p* = 0.0156) (Figure 1F) were analyzed using Spearman’s rank correlation test.

**Figure 2:**
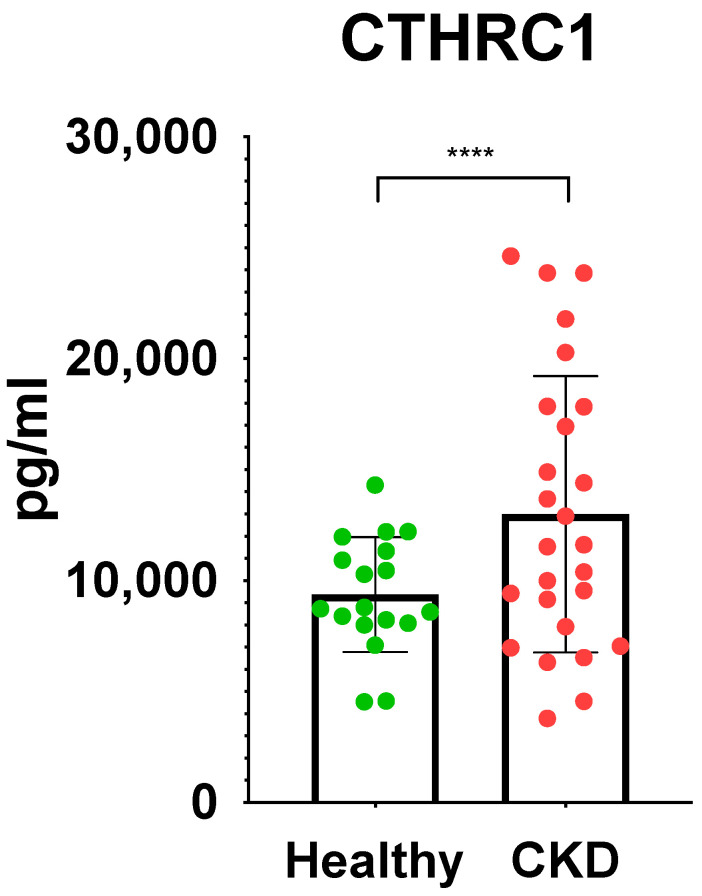
Significant differences in CTHRC1 between control group and CKD patients. Unpaired two-tailed *t*-test was used to calculate the *p* values to define the significance. **** *p* ≤ 0.0001.

**Figure 3:**
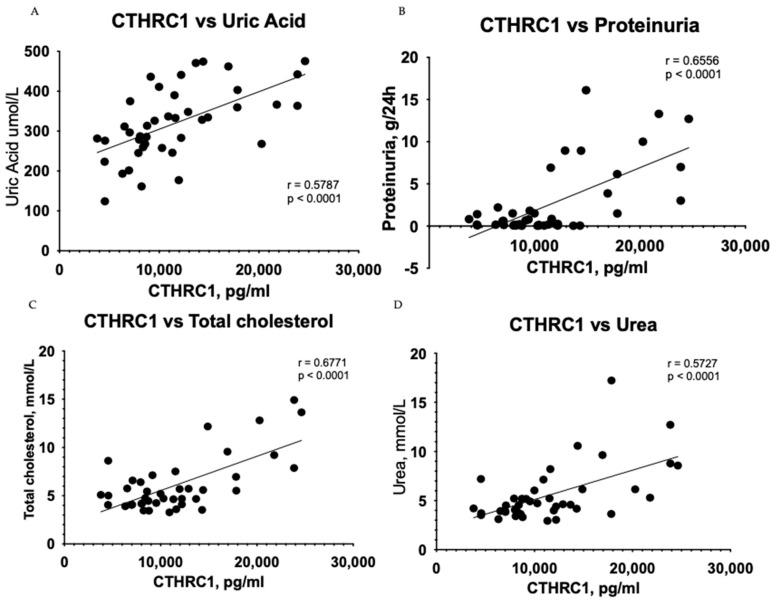

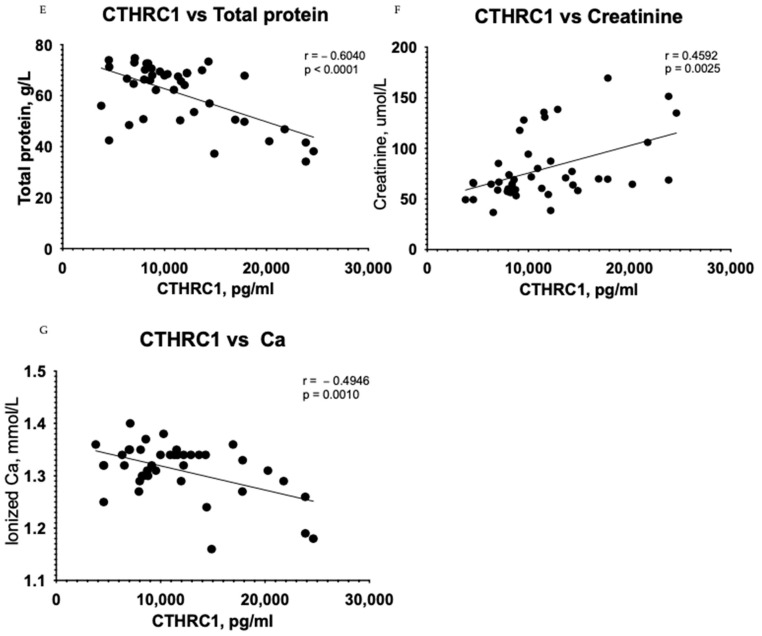
Correlation between plasma levels of CTHRC1 and notable clinical measures in all subjects (Control + CKD groups). The relationship between variables was evaluated using Pearson’s correlation test. Trend lines indicate linear correlation. Pearson’s (r) coefficient and the corresponding *p*-values are shown on each plot. Scatter plot graphs illustrating correlation between (**A**) CTHRC1 and uric acid, (**B**) CTHRC1 and total protein collected in 24 h in urine, (**C**) CTHRC1 and cholesterol, (**D**) CTHRC1 and urea, (**E**) CTHRC1 and plasma total protein levels, (**F**) CTHRC1 and creatinine, (**G**) CTHRC1 and ionized calcium levels.

Urea (*p* ≤ 0.05) and eGFR (*p* ≤ 0.05) values demonstrated adequate significant difference (Table 1), while CTHRC1 showed strong significance (*p* ≤ 0.0001) (Figure 2) between the CKD and control groups. Creatinine levels (*p* ≤ 0.01) (Table 1) were also noteworthy, confirming higher levels in CKD patients than in the control group. Respective correlations were measured together in all subjects (Control + CKD groups) between CTHRC1 and major CKD indicators. We observed similar results in correlation between all subjects with stronger significance between CTHRC1 and CKD indicators (Figure 3).

**Figure 4:**
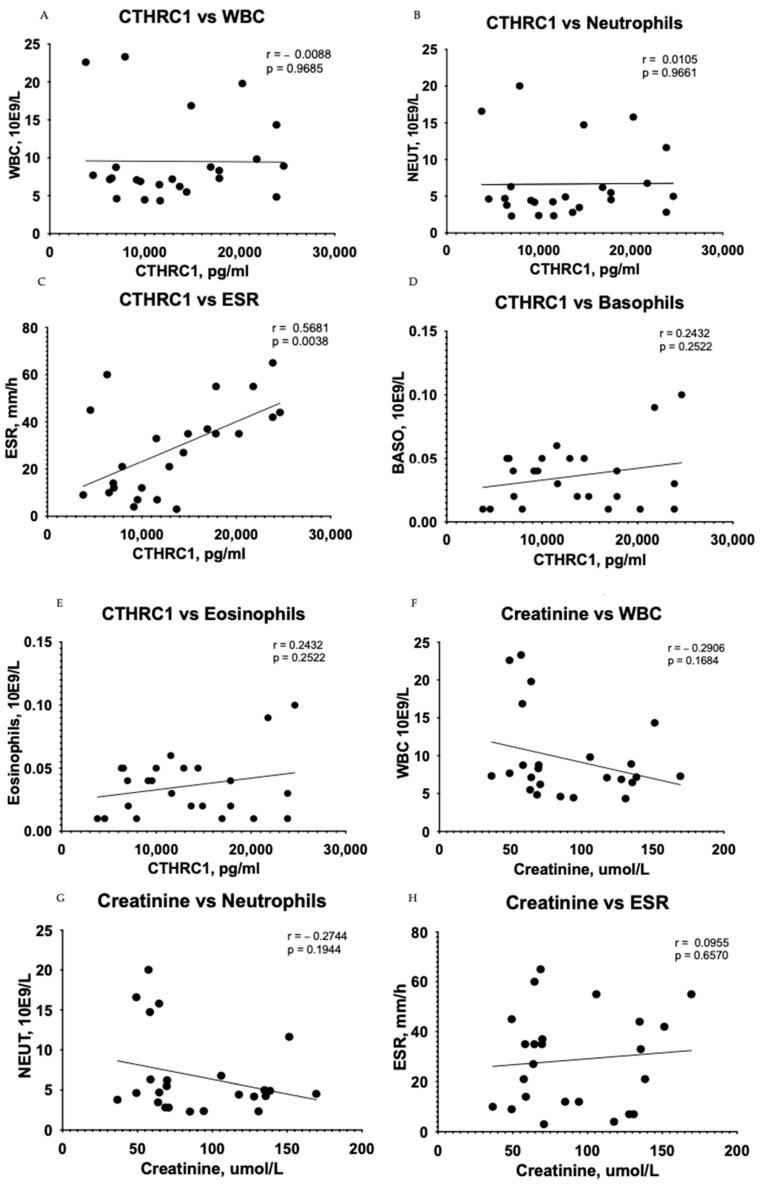

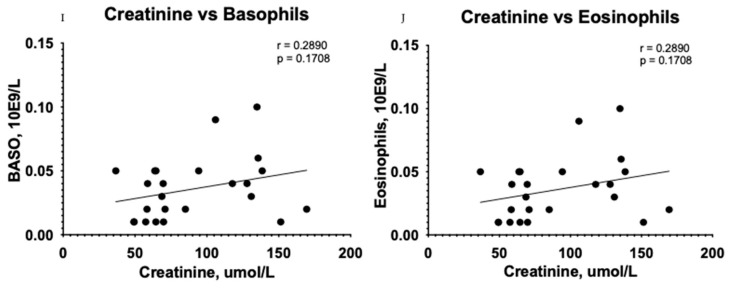
Correlation between plasma levels of CTHRC1, creatinine and blood cells. The relationship between variables was evaluated using Pearson’s correlation test. Trend lines indicate linear correlation. Pearson’s (r) coefficient and the corresponding *p*-values are shown on each plot. Scatter plot graphs illustrating correlation between CTHRC1 and WBCs (**A**), CTHRC1 and neutrophils (**B**), CTHRC1 and basophils (**D**), CTHRC1 and eosinophils (**E**) and CTHRC1 and ESR (**C**) in the CKD group. In addition, similar correlation analysis between creatinine and WBCs (**F**), creatinine and neutrophils (**G**), creatinine and ESR (**H**), creatinine and basophils (**I**), and creatinine and eosinophils (**J**) is displayed. WBC—white blood cells; NEUT—neutrophils.

A correlation test showed subsidiary differences between CTHRC1 and blood cells, including WBCs (r = −0.0088, *p* = 0.9685) (Figure 4A), neutrophils (r = 0.0105, *p* = 0.9661) (Figure 4B), basophils (r = 0.2432, *p* = 0.2522) (Figure 4D), and eosinophils (r = 0.2432, *p* = 0.2522) (Figure 4E) in the CKD group. However, the relationship between erythrocyte sedimentation rate (ESR) and CTHRC1 showed significant correlation (r = 0.5681, *p* = 0.0038) (Figure 4C). In addition, a correlation analysis between creatinine and similar blood cells displayed a complementary tendency (Figure 4H–J).

**Figure 5:**
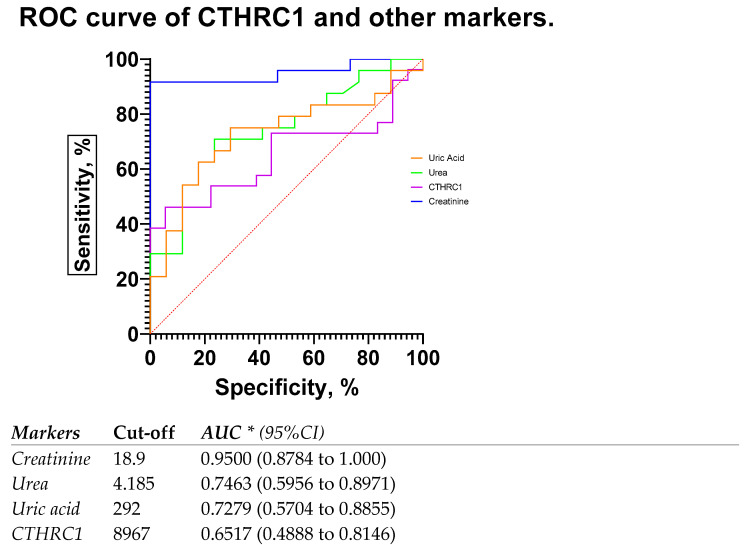
ROC curve and AUC for CTHRC1 levels and other markers in patients with eGFR rates below 60 mL/min/1.73 m^2^ (eGFR < 60) and above 60 mL/min/1.73 m^2^ (eGFR > 60). Cut-off values were defined as the value whose sensitivity and specificity are the closest to the value of the area under the ROC curve. * Area under ROC curve lies with 95% confidence interval.

The most common blood biomarkers were used for comparison with CTHRC1. The use of creatinine or urea measurement to evaluate renal function is justified, since both plasma/serum levels reflect GFR. Regardless of the underlying reason, CKD is linked to a reduction in GFR, and the severity of kidney disease is tightly but adversely correlated with GFR. Thus, we compared eGFR rates in patients with below 60 mL/min/1.73 m^2^ (eGFR < 60) and above 60 mL/min/1.73 m^2^ (eGFR > 60) (Figure 5), where CTHRC1 demonstrated similar AUC scores to other clinical markers.

## 4. Discussion

**Figure 6:**
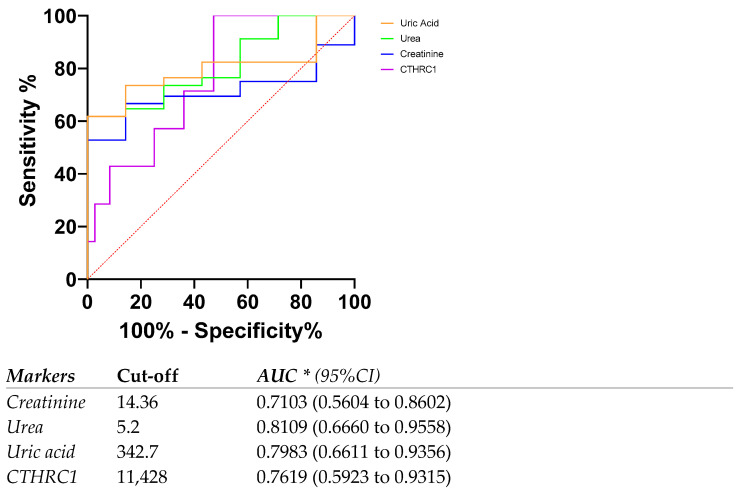
ROC curve and AUC for CTHRC1 levels and other markers in patients with eGFR rates below 60 mL/min/1.73 m^2^ (eGFR < 60). Cut-off values were defined as the value whose sensitivity and specificity are the closest to the value of the area under the ROC curve. * Area under ROC curve lies with 95% confidence interval.

We previously demonstrated the role of CTHRC1 in the migration of fibroblasts in rheumatoid arthritis as well as a potential marker for rheumatoid arthritis disease activity [26,27]. Based on the studies showing the crucial role of TGFβ in the canonical and non-canonical pathways of kidney disease, we hypothesized that CTHRC1 might have a certain role in CKD’s pathogenesis. In this pilot cross-sectional study, we show that CTHRC1 can be a blood-based marker for CKD. We analyzed the levels of plasma CTHRC1 of human subjects to show that elevated CTHRC1 protein can have diagnostic value and can exhibit strong associations with disease severity. Interestingly, the AUC for CTHRC1 levels in patients with eGFR rates below 60 mL/min/1.73 m^2^ (eGFR < 60) was 0.7619 (0.5923 to 0.9315) (Figure 6), whereas for the whole patient group, including eGFR < 60 and >60, the AUC for CTHRC1 was lower compared to creatinine. This indicates that CTHRC1 elevation is proportional to the decline in glomerular filtration capacity. To our knowledge, CTHRC1 was never studied in CKD patients until now. Therefore, further studies are needed to elucidate the functional aspects of CTHRC1 in CKD pathogenesis.

The measurement of blood creatinine concentrations and subsequent determination of the GFR are the foundations of contemporary CKD diagnosis and course monitoring [28]. This method’s drawback is that it performs poorly in terms of early CKD diagnosis. Potential protein biomarkers of CKD have been postulated in a number of studies and assessed in the serum or plasma of patients [29,30,31,32]. Because blood proteins are so accessible, changes in them are frequently observed to help with disease diagnosis. The early stages of the disease can also be diagnosed by changes in blood components. This is particularly true for those with CKD, as changes in renal filtration cause considerable changes in blood composition. For instance, CKD patients have higher serum levels of urea and creatinine, an electrolyte imbalance, and blood clotting. In the present study, we investigated the correlation between CTHRC1 levels and kidney indicators (proteinuria, creatinine, uric acid and urea). It has been reported previously that the CTHRC1 correlates with kidney functions during renal cancer progression; our study revealed that CTHRC1 can be a sensitive, reliable, and more affordable marker for kidney functions. Indeed, using CTHRC1 as a marker for kidney function in clinical practice is suggested by the substantial connection between CTHRC1 and kidney indicators as well as the significant variations in CTHRC1 between the control group and CKD patients.

Although there are many evidences showing an association of WBC and other inflammatory markers with kidney function [16,33], there are also inconsistent reports showing fair function in predicting the risk of kidney dysfunction (Refs. [16,33]). In our study, despite the significant differences in WBC and neutrophils between the CKD group and control group, we did not observe any significant correlations between WBC (including neutrophils, eosinophils, basophils) and CTHRC1. The same pattern of correlations was obtained with creatinine, and the results were consistent with CTHRC1 (Figure 1). We suspect these phenomena need to be clarified with a larger sample size and with an inclusion of CKD patients with later stages (IV–V).

In this study, CTHRC1 was detectable in the control group. Therefore, understanding the threshold for the circulating protein in the healthy status is important for the best sensitivity/specificity ratio in pathology. In the study of Duarte et al., CTHRC1 was measured in 1300 patients with various diagnoses and 40 healthy volunteers, and approximately one-third of assayed plasma samples was negative. The CTHRC1 levels in 80% of healthy volunteers were below the detection limit (160 pg/mL) and elevated in 20% of individuals with red hair and fair skin. The median concentration of CTHRC1 for healthy volunteers was 69.6 ng/mL [34]. This value is almost three times higher than the mean value that we obtained in this study (max value: 15 ng/mL, mean value: 9 ng/mL). In our previous study, healthy people showed a presence of CTHRC1 in circulation maximum of 25 ng/mL [27], and this indicates a good agreement between our current study and published reports. We observe a significant negative correlation between eGFR and CTHRC1 (*p* = 0.0366) (Figure 1H). It is well established that eFGR reduction and proteinuria increase are risk factors for CKD. Our findings suggest that CTHRC1 significantly correlates with eGFR values alongside with other CKD indicators. During CKD progression, myofibroblasts produce collagens, specifically collagen type I and III, which consequently contributes to kidney fibrosis [35,36]. The decline in GFR is mainly the result of tubulointerstitial fibrosis and mesangial expansion, which are caused by the elevation of inflammatory cytokines and extracellular matrix (ECM) deposition [37]. Active mesangial cells secrete chemokines and cytokines, which will eventually have a huge impact on local glomerular cells as well as on leukocytes. During chronic mesangial cell activation, ECM deposition grows gradually, leading to interstitial fibrosis and eventually to glomerulosclerosis [38]. The accumulation of ECM in the tubulointerstitium starts with NF-kB activation and an upregulation of TGFb. While NF-kB controls chemokine and cytokine production by regulating proinflammatory genes, TGFb expression promotes the transformation of mesangial/epithelial cells to fibroblasts and myofibroblasts [35,39,40].

TGFb regulates the expression of CTHRC1 in many signaling pathways, including TGF-β, Wnt, integrin β/FAK, PI3K/AKT/ERK, and PKC-δ/ERK signaling pathways [41,42].

There are many studies showing the relationship between the TGFb family and CTHRC1, such as the induction of CTHRC1 transcription and expression by TGFb and BMP in NIH3T3 cells [9]. During TGFb signaling, the Smad2/3-Smad4 complex accumulates in the nucleus and causes collagen type I deposition. This process can be caused by the binding of phospho-Smad3 to the promoter with the subsequent activation of CTHRC1 transcription [43]. It was also reported that CTHRC1 can downregulate TGFb expression at late phases of wound healing [42]; also, there are studies showing that TGFb and CTHRC1 interactions occur in a concentration-dependent manner, i.e., the overexpression of CTHRC1 can have a negative effect on collagen synthesis by accelerating the proteosomal degradation of phospho-Smad3 [44]. These observations need further clarifications on CKD pathogenesis.

CTHRC1 inhibits collagen deposition and promotes cell migration by preventing Smad2/3 phosphorylation in TGF-β signaling [10]. CTHRC1 is a positive regulator of the non-canonical Wnt/PCP pathway, which is implicated in the regulation of cell motility, carcinogenesis, and chondrocyte maturation in developing cartilage. CTHRC1 interacts with Fzd5 and Fzd6 and promotes the formation of the Ror2/Fzd/Wnt complex and downstream RhoA/Rac1 phosphorylation. Thus, CTHRC1 enhances PCP-Wnt signaling and inhibits the canonical Wnt-β-catenin pathway [16]. At the current level of understanding, CTHRC1 might have a role in renal fibrosis, as it inhibits the TGFβ/Smad pathway. Although the role of TGFβ/Smad signaling was proven to be crucial in renal fibrosis and inflammation, clinical trials showed unsatisfactory results in a blockade of upstream TGFβ signaling [45]. The blockade of TGFβ signaling reduces renal fibrosis, but on the other hand, it can initiate renal inflammation and can cause unexpected renal injuries [41]. In the Wnt/β-catenin pathway, Wnt molecules form a complex with Frizzled (Fzd) receptors and co-receptors LRP 5/6. This interaction leads to the accumulation and translocation of β-catenin into the nucleus and triggers the transcription of Wnt target genes [46,47]. β-catenin mediates the fibrogenic signaling pathway by integrating TGF-β/Smad, integrin/ILK, the Wnt/β-catenin pathway, and the renin–angiotensin system (RAS) [46,48]. The RAS has a crucial role in blood pressure regulation, in controlling renal blood flow, and in glomerular filtration rate as well as tubular sodium chloride levels and water transport mechanisms [11]. Wnt/β-catenin controls the expression of multiple RAS genes, and RAS in turn induces multiple Wnt genes, promoting kidney damage [49,50,51]. Yamamoto et al. showed that CTHRC1 stabilizes the Wnt/Fzd/LRP5/6 complex [16]. It was also reported that CTHRC1 is expressed in adventitial fibroblasts and neo-intimal smooth muscle cells and promotes cell migration by decreasing ECM deposition; it also has a crucial role in tissue repair after injury [9]. Fibroblast proliferation and differentiation to myofibroblasts by activation of the Wnt/β-catenin pathway is the main driving force in renal fibrosis [52,53]. It was shown that CTHRC1 is highly expressed in renal cell carcinoma tissue, and the knockdown of CTHRC1 was shown to inhibit the proliferation of carcinoma cells as well as negatively affect the epithelial–mesenchymal transition process. By inhibiting β-catenin expression in renal cell carcinoma tissues, cell migration and invasion processes were suppressed [54].

To our knowledge, CTHRC1 was studied in CKD patients for the first time, and there is a lack of information regarding the role of this protein in CKD pathogenesis. Further studies would be required to elucidate the exact function in glomerular filtration and reabsorption. Based on the studies showing the significant role of Wnt/B catenin pathways, including ligands and receptors in CKD progression, we hypothesize that CTHRC1 should have an essential role as well.

Numerous restrictions apply to our study, particularly, the limited sample size in each group, and due to its cross-sectional form, it is challenging to pinpoint the connection between CTHRC1 and the early-stage of CKD as well as the progression of the disease. To determine CTHRC1 expression in relation to disease stage, a large number of patients with stages from 1 to 5 should be included, and a longitudinal study would be necessary. Medical history, including the etiology of CKD and any current medications, was not reported in the current study.

To further the obtained results, the effect of CTHRC1 to podocyte detachment and on fibroblast transformation to myofibroblasts as well as exact role of this protein in scar formation should be studied. This study is the second study in the Kazakhstani population where CTHRC1 is investigated; therefore, the threshold for CTHRC1 should be established for the Kazakhstani population.

## 5. Conclusions

The study’s findings have shown that declines in kidney function are linked to an elevation of CTHRC1 levels in CKD patients. At the current level of understanding, elevated plasma CTHRC1 levels may indicate disease status in CKD, and these findings warrant confirmation in a larger, more comprehensive patient population. Our study demonstrated that CTHRC1 has a significant correlation with creatinine, total protein, urea, and uric acid, which are considered to be the main indicators of CKD and its progression. Therefore, we identify CTHRC1 as a potential marker of CKD, which can improve the diagnosis.

## Figures and Tables

**Table 1:** Notable clinical characteristics of the study population.

Characteristics *	CKD (*n* = 26)	Control Group (*n* = 18)	*p*-Value
**Age—years**	38.8 ± 14.5	34.7 ± 8.05	0.2826
**Male—no. (%)**	16 (61.5)	5 (27.8)	N/A
**Female—no. (%)**	10 (38.5)	13 (72.2)	N/A
24 h proteinuria—g/24 h **	1.66 (0.75–7.49)	0.09 (0.06–0.17)	**0.0006**
Semi-quantitative protein—g/L **	2.59 (0.75–5)	0	**<0.0001**
**eGFR, ml/min/1.73m^2^**	91.12 ± 34.2	109.76 ± 12.4	**0.0279**
**Stage I—no. (%)**	13 (50)	N/A	N/A
**Stage II—no. (%)**	7 (26.92)	N/A	N/A
**Stage III—no. (%)**	6 (23.07)	N/A	N/A
Complete blood count *			
**WBC—10^9^/L**	9.52 ± 5.29	5.38 ± 0.99	**0.0059**
Lymphocytes—10^9^/L **	1.9 (1.56–2.48)	1.6 (1.34–2.00)	0.3331
**Hemoglobin—g/L**	129.9 ± 24.3	133.25 ± 20.8	0.6557
**RBC—10^12^/L**	4.55 ± 0.66	4.79 ± 0.52	0.2268
Platelets—10^9^/L **	297 (223.5–363.8)	255 (229.3–292.3)	0.2162
**Monocytes—10^9^/L**	0.63 ± 0.32	0.40 ± 0.1	**0.0068**
**Neutrophils—10^9^/L**	6.66 ± 5.06	3.13 ± 0.82	**0.0089**
**Eosinophils–10^9^/L**	0.15 ± 0.13	0.11 ± 0.1	0.4357
**Basophils–10^9^/L**	0.03 ± 0.02	0.04 ± 0.02	0.4375
**Neutrophils—10^9^/L**	6.66 ± 5.06	3.13 ± 0.82	**0.0089**
**Immature Granulocytes—10^9^/L**	0.05 ± 0.08	0.008 ± 0.007	0.1570
Erythrocyte sedimentation rate—mm/h **	30 (10.5–43.5)	10 (5.25–14.25)	**0.0024**
Biochemical analysis of blood plasma *			
**Uric acid—umol/L**	346.7 ± 95.1	282.3 ± 66.9	**0.0214**
**Creatinine—umol/L**	90.55 ± 38.1	64.98 ± 11.5	**0.0111**
LDLs—umol/L **	6.53 (2.83–6.53)	2.76 (2.17–3.20)	**0.0015**
Urea—mmol/L **	5.22 (4.06–8.48)	4.06 (3.53–4.64)	**0.0178**
**Total protein—g/L**	54.4 ± 11.8	69.4 ± 3.5	**<0.0001**
**Total cholesterol—mmol/L**	7.2 ± 3.2	4.4 ± 0.8	**0.0016**
**Triglycerides—mmol/L**	2 ± 0.99	0.96 ± 0.53	**0.0001**
**Albumin—%**	53.9 ± 11.7	67.2 ± 3.5	**<0.0001**
**Alpha-1-Globulin—%**	2.93 ± 0.7	1.85 ± 0.3	**<0.0001**
**Alpha-2-Globulin—%**	17 ± 8.1	8.4 ± 0.9	**<0.0001**
**Beta-1-Globulin—%**	8.7 ± 2.45	6.1 ± 0.82	**0.0002**
**Beta-2-Globulin—%**	5.8 ± 2.46	3.36 ± 0.53	**0.0003**
**Ionized Ca—mmol/L**	1.29 ± 0.06	1.33 ± 0.03	0.0548

* Average values with standard deviation (±SD) are presented. N/A—parameter not determined. ** Medians with 25% and 75% interquartile ranges (LQ-UQ) are presented. Na—Sodium; eGFR—Estimated glomerular filtration rate; LDLs—Low-density lipoproteins; Ca—Calcium; WBC—White blood cells; BLD—Blood, erythrocyte per µL; Pro—Total protein; N/A—Not determined; CKD—Chronic kidney disease.

## Data Availability

The data presented in this study are available on request from the corresponding author. The data are not publicly available due to privacy and ethical regulations.
